# A Novel Surface Plasmon Resonance Biosensor for the Rapid Detection of Botulinum Neurotoxins

**DOI:** 10.3390/bios7030032

**Published:** 2017-08-07

**Authors:** Kruti Patel, Shmuel Halevi, Paul Melman, John Schwartz, Shuowei Cai, Bal Ram Singh

**Affiliations:** 1 Department of Chemistry and Biochemistry, University of Massachusetts Dartmouth, 285 Old Westport Road, North Dartmouth, MA 02747, USA; kruti.vasa@gmail.com (K.P.); scai@umassd.edu (S.C.); 2 Newton Photonics, Newton, MA 02458, USA; shalevi20@gmail.com (S.H.); melmanp@gmail.com (P.M.); 3 AcuityBio, Inc., 200 Upland Rd., Newton, MA 02460, USA; jay@acuitybio.com; 4 Botulinum Research Center, Institute of Advanced Sciences, Dartmouth, MA 02747, USA

**Keywords:** biosensor, botulinum, clostridium, rapid detection, surface plasmon resonance

## Abstract

Botulinum neurotoxins (BoNTs) are Category A agents on the NIAID (National Institute of Allergy and Infectious Diseases) priority pathogen list owing to their extreme toxicity and the relative ease of production. These deadly toxins, in minute quantities (estimated human i.v. lethal dose LD_50_ of 1–2 ng/kg body weight), cause fatal flaccid paralysis by blocking neurotransmitter release. The current gold standard detection method, the mouse-bioassay, often takes days to confirm botulism. Furthermore, there are no effective antidotes known to reverse the symptoms of botulism, and as a result, patients with severe botulism often require meticulous care during the prolonged paralytic illness. To combat potential bio-terrorism incidents of botulinum neurotoxins, their rapid detection is paramount. Surface plasmon resonance (SPR) is a very sensitive technique to examine bio-molecular interactions. The label-free, real-time analysis, with high sensitivity and low sample consumption makes this technology particularly suitable for detection of the toxin. In this study, we demonstrated the feasibility in an assay with a newly designed SPR instrument for the rapid detection of botulinum neurotoxins. The LOD (limit of detection) of the Newton Photonics (NP) SPR based assay is 6.76 pg/mL for Botulinum Neurotoxin type A Light Chain (BoNT/A LC). We established that the detection sensitivity of the system is comparable to the traditional mouse LD_50_ bioassay in BoNT/A using this SPR technology.

## 1. Introduction

Botulinum poisoning is a serious and well-recognized biothreat. Botulinum neurotoxins (BoNTs; seven known serotypes, A–G)) are extremely potent, relatively simple to prepare and disburse, and have already been used as an agent of bioterror [1]. BoNTs are zinc metalloproteases comprising a 50 kDa light chain (LC) and a 100 kDa heavy chain (HC). The LC is the proteolytic domain of the BoNT that cleaves one of the soluble N-ethylmaleimide-sensitive factor activating protein receptor (SNARE) target proteins at the presynaptic termini, leading to inhibition of the neurotransmitter release. The HC plays an accessary role in binding to nerve cells and the translocation of the LC into the cytosol of nerve cells. Due to their high potency and lack of countermeasures, BoNTs are classified as Category A biothreat agents, and are placed under Tier 1 Select Agents by the US Centers for Disease Control [2,3].

Currently, the gold standard laboratory diagnosis of botulism is based on the mouse bioassay, which is slow (2–4 days), and requires dedicated laboratory settings, and trained personnel. This underscores the unmet need for a BoNT technology with comparable sensitivity that provides a short time to results and is easy to use. BoNT serotypes A and B (BoNT/A, BoNT/B) are responsible for the most cases of fatal intoxication in humans, and thus have high potential for being used as biological weapons [4].

The mouse bioassay is currently the only accepted method to confirm the detection of BoNTs [5]. The sensitivity of a mouse bioassay is 1 MLD (injection volume is 0.5 mL), or about 20–30 pg of BoNT/A [6]. The mouse bioassay has been considered the “gold standard” for the assessment of botulinum detection. However, there are several shortcomings associated with mouse bioassay: mice can die non-specifically during the process, the test takes about four days to complete, the process is labor intensive and requires a special animal facility, and highly trained and immunized personnel are required to carry out the test [7]. The mouse bioassay is not suitable for routine quantification of samples and cannot meet the required testing capacity in the event of a real biodefense deployment, because of the large number of animals needed to obtain statistically significant results [8].

Several new methods for the detection of BoNT have been developed, including ELISA (enzyme-linked immunosorbent assay), electro-chemiluminescence, endopeptidase-mass spectrometry (endopep-MS), immuno-polymerase chain reaction (immuno-PCR), and most recently, the protease activity assay of BoNTs [9,10,11,12,13]. The sensitivity of conventional ELISA is between 10–100 MLD/mL [10,13]; the sensitivity of immuno-PCR and electro-chemiluminescence methods can reach or even surpass the sensitivity of mouse bioassay [10,14]. Both methods, however, suffer from some significant shortcomings that prevent them from being widely used as standard assays. Immuno-PCR requires thermal cycling and analysis that restricts its use for high-throughput screening and the appearance of major background noise also limits immuno-PCR as an effective assay. The electro-chemiluminescence assay suffers from interference from sample matrices, has an inherent narrow dynamic range, and requires an expensive specialized laboratory [10].

Some the newly developed assays based on the endopeptidase activity of BoNT are very promising as these assays could incorporate measurement of the biological activity of the neurotoxins. An assay, based on immunoaffinity to capture the toxin followed by an endopeptidase activity assay, has been shown to have sensitivity in the range of the mouse bioassay [15,16,17,18]. This assay, however, has been only partially successful so far, as it lacks multiplexing and high throughput capacity, and it involves multiple biochemical steps, requiring additional time and equipment. Other endopeptidase activity-based assays have not matched the mouse bioassay in sensitivity [19,20,21], mainly due to the limited sensitivity of a particular ELISA assay, or the specific affinity of the primary antibodies used to distinguish cleaved from uncleaved peptide [19,20,21]. In general, biosensors have provided rapid detection methods for biological toxins, and have been applied to the detection of BoNTs [22,23]. While the testing time can be as short as 10 min, the detection limit of these biosensor-based methods has been at the ng/mL level for BoNTs using the Biacore SPR system [17].

A previous study [24] has demonstrated the use of an SPR sensor for the sensitive detection of botulinum activity. In this study, synaptic vesicles (SV) naturally studded with synaptosomal-associated protein 25 (SNAP-25) and vesicle associated membrane protein (VAMP) were prepared from cells and exposed to BoNT/B and BoNT/F in vitro. The cleavage event was detected by a standard SPR instrument (Biacore) at the sub-picogram level using surface-bound antibodies directed at the C-terminus of the target proteins [24]. This experiment was useful in proving the feasibility of sub-picogram BoNT detection using an endopeptidase assay on an SPR sensor. However, this assay is cumbersome and the reagents are difficult to prepare (brain tissues, SV, antibodies), and it requires animal husbandry and trained personnel for use in a commercial screening assay.

To address several of the above issues, we set the goal to build a sensor for rapid (30~60 min) in vitro detection of BoNT/A and BoNT/B LC with sensitivity comparable to the mouse bioassay (~20–30 pg/mL), for use in diagnostic laboratories and public health facilities [6]. We have developed both a more sensitive and robust SPR sensor and a better substrate for the endopeptidase assay. We used the LC as the surrogate for the assay development since it is the toxic domain of BoNT. The combination of the unique SPR system and a more suitable substrate resulted in a better detection assay of BoNT/A activity, with a sensitivity comparable to that of the mouse bioassay.

## 2. Material, Instrument Design, and Methods

The maleimido gold was purchased from Nanoprobes, (Yaphank, NY, USA); 10X PBS buffer was obtained from Thermo Fisher Scientific (Waltham, MA, USA); D_2_O was purchased from United Nuclear (East Lansing, MI, USA); and neutravidin was obtained from Pierce Biotechnology, (Rockford, IL, USA).

### 2.1. Design of the Waveguide-SPR Sensor

A key goal of this project was to build an engineering prototype of the SPR sensor, including an optical chip reader and a batch of SPR chips.

**Figure 1:**
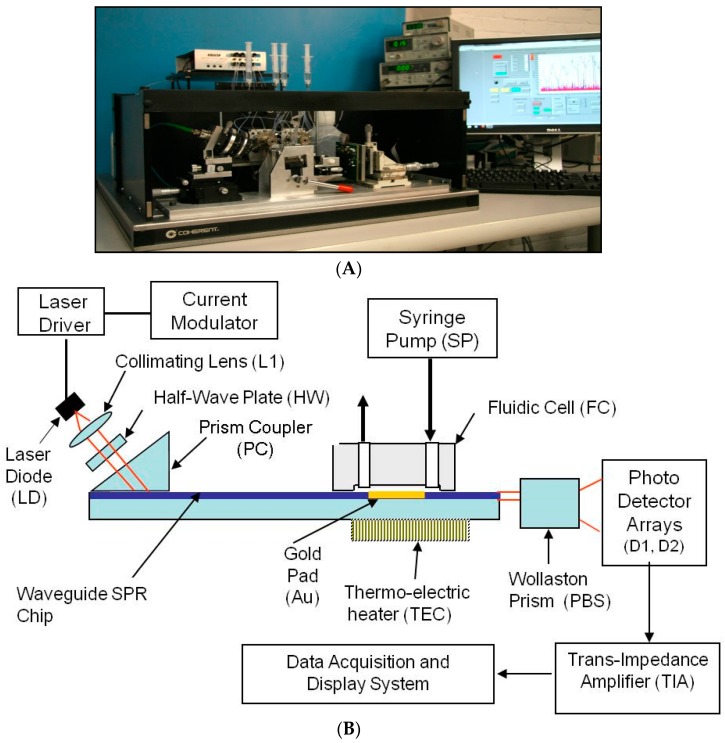
(**A**) Engineering prototype of the four-channel SPR reader; (**B**) Waveguide SPR reader diagram.

The diagram of the SPR reader, in Figure 1, shows the mechanical configuration, optical system, a portion of the liquid handling subsystem, and the computer monitor. The LabView™ program (National Instruments, Austin, TX, USA) running on a standard PC was used to acquire and process the electronic signals. It implements a differential detection algorithm, displays the SPR sensorgrams and records the test results.

The Newton Photonics (NP) proprietary waveguide-SPR sensor detects intensity changes in the light beam transmitted by an optical waveguide. In contrast to the conventional SPR approach, where the molecular interaction (cleavage or binding) is detected by measuring the change in the resonance angle, detection in the waveguide-based sensor is performed by measuring the change in intensity of the TM-polarized (transverse magnetic) component of a transmitted light beam (probe beam). The high sensitivity is achieved by deploying a second, TE-polarized (transverse electric) reference beam. TM and TE are the two propagation modes of the light beam in the waveguide. Only the TM mode induces an SPR response, and therefore the TE mode can be used as a reference in the differential detection scheme. The reference beam is generated by the same light source and propagates along the same path as the probe beam, but does not couple to the surface plasmon wave. As the surface-bound biomolecular mass changes, the SPR resonance curve starts to shift, causing a change in intensity of the TM beam while leaving the TE beam unaffected. Multi-channel operation was achieved by converting the collimated input light beam into a line that is oriented parallel to the SPR chip surface, and passing the light that emanated from the chip through a four-slot comb-shaped mask. The four output beams are then split to their TM and TE polarization components by a Wollaston prism and the signal intensities of each polarization state are measured by two photodetector arrays.

A differential SPR signal is formed by measuring the real-time difference between the intensity of the probe beam and the reference beam (TM − TE) in each optical channel and then dividing the result by the total light intensity (TM + TE) to normalize for the power fluctuation of the light source. This signal is uncorrupted by the mechanical and electronic noise present in all SPR instruments.

**Figure 2:**
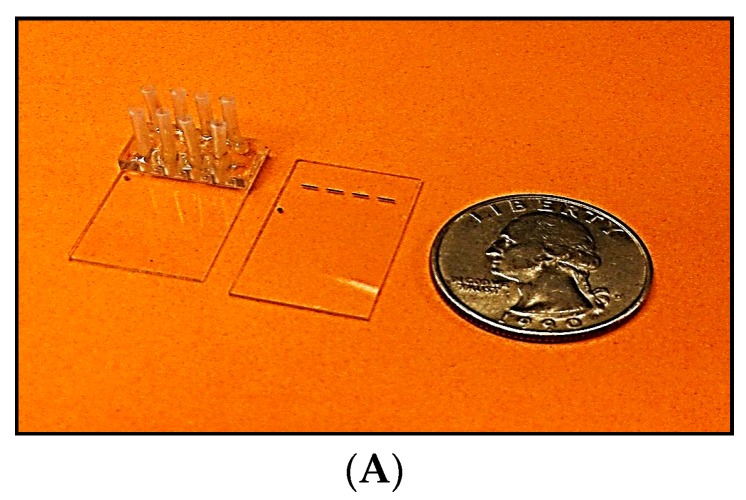

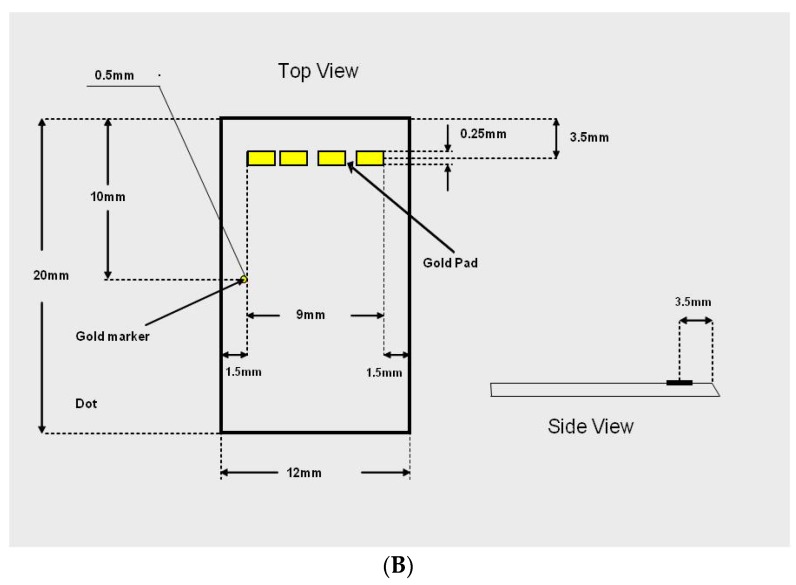
(**A**) Four-channel SPR chip and integrated flow cell [Coin is shown for reference]; (**B**) Zoomed version of the layout of a multi-channel glass-based waveguide SPR chip.

The four-channel SPR chip, comprising a glass-based planar waveguide and four gold pads (shown in Figure 2), was fabricated according to NP specifications, using a conventional photo-lithographic process. A batch of 50 chips was produced from a single 8” glass wafer. These chips can be mass-produced at low cost (<$2) in a conventional optical foundry. Of these, 20 chips were fitted with precision glass flow cells (made by CiDRA, Wallingford, CT, USA). Subsequently, input and output ports Teflon tubes, (IDEX Health & science LLC, Oak Harbor, WA, USA) were inserted and secured in place by epoxy resin. Each assembled chip was then mounted on a steel cradle that was inserted into the SPR reader and set in place by a mechanical locking mechanism.

The unique NP SPR-waveguide sensor exhibits several advantages over conventional SPRs:

1. Compared to many waveguide sensors described in the literature, it is one of the most sensitive (single mode configuration) and the only sensor that can have its chips produced by standard techniques of photolithography and plasma deposition.
2. Relative to the Kretschmann configuration (e.g., Biacore) it is substantially less expensive (Table 1). Adding the cost of labor, testing and marketing, the price of this instrument should not exceed $30,000. Moreover, dextran coated chips for Biacore cost $150 per chip, compared to the NP SPR chips which can be mass produced at less than $2 per chip because of the photolithography technology to define the sensing pads.

### 2.2. Waveguide Reader Design

The SPR reader is the instrument that measures the refractive index changes at the chip surface caused by changes of the surface-bound molecular mass. These changes can be due to binding or cleavage reaction on the chip surface. Figure 1B shows the design of the SPR reader.

The laser diode (LD) emits up to 10 mW at 630 nm. The laser light is modulated at a frequency of 900 Hz using current modulation. The orientation of the polarization plane is adjusted to be 45° with respect to the chip surface by means of a half wave-plate (HW), such that half of the light is polarized parallel to the chip surface (TE mode) and half perpendicular to it (TM mode). The 45° polarized beam is coupled to the waveguide through a high index prism (PC) and the two orthogonally polarized beams propagate under the sensing gold pad (Au). At the output of the waveguide the light beam is split into two polarization components by means of a Wollaston prism (PBS). The optical signals are captured and converted to electronic signals by two linear photo-detector arrays and low-noise trans-impedance amplifiers (TIA). The signal acquisition, conditioning and manipulation are performed digitally using the National Instruments virtual instrument hardware and software.

Multiple detection channels are implemented by illuminating the chip with a laser beam projected through a line generator onto the surface of the chip, and placing a comb-shaped mask in the optical path at the output end of the waveguide. This way the full length of the gold pad is activated and each opening in the mask forms a differential detection channel. Each optical channel is supported by two photo-detector cells (one in each photo-detector array), and two TIAs for the TM and TE polarization components of the light beam. This optical design allows the formation of up to eight differential detection channels on a 12 mm-wide SPR chip without introducing cross-talk between adjacent channels.

### 2.3. Waveguide SPR Chip

The SPR chip is a glass substrate with a planar waveguide embedded on its top surface. Light coupled to the waveguide is confined to the very thin guiding layer. A thin gold film is deposited on the guiding layer. The guided light propagates underneath the gold surface and the TM polarization excites surface waves in the gold film. This process of excitation reduces the transmitted light intensity (energy is absorbed in the gold film) in relation to the index of refraction of the layer adjacent to the film surface. The SPR chips was fabricated in an optical foundry (ANDevices, Fremont, CA, USA), in a standard optical chip production process.

The variables that define the performance of an SPR chip are the waveguide parameters and the composition, thickness and length of the sensing gold pad. The key design parameters are listed below in Table 2. The chip layout has been shown in Figure 2A,B indicates the design of the chip.

### 2.4. Liquid Handling System

The fluidic subsystem comprised of a four-channel syringe pump (SP), a degasser unit, five-port injection valve, three selector valves, and a four-channel flow cell (FC). The flow cell was fabricated by casting a two-part silicone compound (General Electric RTV-615) in a specially designed aluminum mold with a glass bottom. A relief pattern of the flow channels was created by thick photo-lithography process on a glass plate (Intelligent Micro Patterning, St. Petersburg, FL, USA). The glass plate was attached to an aluminum enclosure, fitted with 23-gauge needles to form the input and output ports of the flow cell. Subsequent to the casting, the aluminum enclosure was machined and used as a flow-cell mount in the SPR system. The design and fabrication methods and materials of the flow cell were successfully developed.

The difference between the single-channel and the multi-channel flow cell is the number and orientation of the flow channels. In the single-channel instrument the flow channel runs along the full length of the gold pad. In the multi-channel design the flow channels traverse the gold pad. Using external valves, serial or parallel flow configurations can be selected, thus supporting selective or common immobilization and detection functions without removing the flow cell from the SPR chip.

### 2.5. Heavy Water Experiment

Pure water was ten times serially diluted with deuterated heavy water with a concentration of 2.2%, 0.22% and 0.022%. The samples were injected in the SPR. The SPR sensorgram of the pure water (blank) was compared with different concentrations of heavy water.

### 2.6. Regeneration of Gold Surface

The regenaretion of the chip gold surface was carried out with the injection of 10 mM NaOH and 1% Triton X-100 for 30 min (two runs for 15 min each). This was followed by the wash of the suface with PBS for 30 min for the best regeneration of the surface.

### 2.7. BoNT/A LC and BoNT/B LC Purification

Recombinant BoNT/A LC and BoNT/B LC were purified using Ni^2 +^ column according to the method described earlier [25,26]. BoNT/A and BoNT/B light chains were purified in phosphate buffer (10 mM sodium phosphate, pH 8.0, containing 150 mM NaCl).

### 2.8. Design of Cleavage Substrates for SPR Botulinum Assay

**Figure 3:**
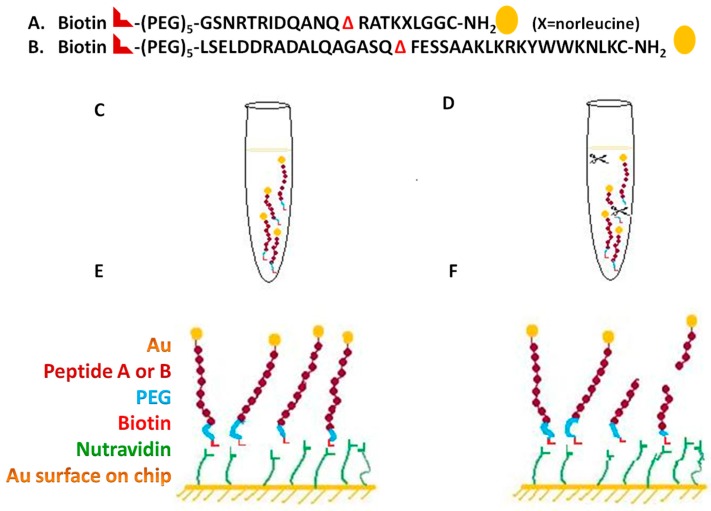
Schematics of the endopeptidase assay design for SPR. The change in the reflective index directly corresponds to the change in mass of the peptide substrate at the chip surface. (**A**) Indicated substrate sequence for the botulinum neurotoxin A, the peptide A incorporated with the cleavage site Q–R, (Δ represents the cleavage site); (**B**) The indicated substrate sequence for the botulinum neurotoxin B, the substrate incorporated the cleavage site Q–F (Δ represents the cleavage site); (**C**) Control (uncleaved peptide A or B) for the SPR sample included 3pM peptide A or B; (**D**) 3 pM peptide A or B sample spiked with different concentrations of botulinum neurotoxin A or B light chain in the concentration range 6.6 µg/mL to 6.6 pg/mL; (**E**) Corresponded to the control (uncleaved peptide A or B) at the SPR Au chip surface. Biotin binds with the nutravidin at the Au chip surface; (**F**) Corresponds to the sample dose-dependent cleaved peptide samples bound through biotin at the Au chip surface to nutravidin.

The type A and B cleavage substrate peptides were designed, as shown in Figure 3A,B, were synthesized by AnaSpec (Fremont, CA, USA). Each peptide has three key features: biotin at the proximal end, to enable binding to the neutravidin coated SPR chip; NH_2_ group at the other end (the far end from the sensor surface) to enable the attachment of a gold nanoparticle; and a toxin-specific cleavage sequence in the middle (SNAP-25, VAMP).

Cleavage by the toxin separates the biotin-tipped segment from the rest of the molecule, thus enabling SPR detection of toxin activity based on the mass difference between the cleaved vs. uncleaved surface-bound peptide as shown in Figure 3.

The substrate used for BoNT/A was:

Biotin-(PEG)_5_-GSNRTRIDQAN**Q** Δ**R**ATKXLGGC-NH_2_
(X = 2-aminohexanoic acid (norleucine)). BoNT/A specifically cleaves SNAP-25, a membrane protein with a cleavage site Gln197–Arg198 (ΔQ and R), Δ represents the cleavage site.

The key feature in the peptide is the use of a 150 kDa gold nanoparticle instead of an antibody at the distal end of the peptide. While of similar molecular mass, the gold nanoparticle (1.4 nM diameter) is more compact than the antibody and prevents steric hindrance to the cleavage reaction. Moreover, the gold nanoparticle is far more stable than the antibody, thus extending the shelf life of the substrate and eliminating refrigerated storage requirements.

For the labeling of the peptide with gold nanoparticles, maleimido gold (purchased from Nanoprobes, Yaphank, NY, USA) was used. The cysteine was covalently linked to a 1.4 nm monomaleimido-nanogold particle. Monomaleimido-nanogold is a gold cluster of about 50 kDa with an organic shell and a monofunctional reactive arm with a maleimide (which reacts with thiols). The labeling and storage of the peptides was performed according to the manufacturer’s directions [27]. Briefly, a 5:1 molar ratio of maleimido-gold nanoparticle over peptide was used, and the reaction was carried out in 1 mL of 20 mM sodium phosphate buffer (pH 6.5) with 150 mM sodium chloride at room temperature for 3h. Au-labeled peptides were aliquoted and stored frozen at −20 °C until use without further purification. The ratio of gold-nanoparticle and peptide in the labeled conjugate was 1:1.

### 2.9. Detection of BoNT/A LC by NP Waveguide SPR System

The SPR chip was loaded onto the reader, locked in place, and attached to the fluidic harness. The chip was prepared for detection by cleaning with 0.12 N NaOH + 1% Triton X-100, followed by neutravidin immobilization (50 µg/mL in 1X PBS). These preparations can be carried out in advance of the detection session, and ultimately, pre-coated chips can be used. The SPR sensorgram show the SPR signal intensity in refractive index units (RIU) at each active channel as a function of time (seconds). The real time SPR measurements were recorded as RIU signal vs. time reflecting binding of the substrate peptide. The recording carried for 20 min to reach steady state binding. The flow rate in the preparation stage, as well as the detection stage, was 40 µL/s, and the sample loop volume was 400 µL. Thus, the sample flow time over the chip was 10 min. The total run time was around 20 min for the light chain samples. The run time was extended in order to ensure washing of the chip. In the future, total detection time can be substantially reduced by increasing the flow rate and terminating the session on the basis of positive response after the sample flow time.

BoNT/A or BoNT/B light chain samples ranging 6.6 µg/mL to 6.6 pg/mL in concentration were incubated for 30 min at 37 °C with 3 pM of peptide A or peptide B, respectively, in 20 mM Hepes buffer, pH 7.0, containing 1 mM DTT, 1 mg/mL protease free BSA, 60 µM ZnCl_2_. The reaction was terminated by adding 1650 µL PBS 7.4, and 150 mM NaCl, pH 7.4. Since the SPR signal intensity is proportional to the surface-bound molecular mass, the intensity ratio of the sample channel (cleaved peptide) to the control channel (uncleaved peptide) is proportional to the BoNT/A LC activity in the sample.

The limit of detection (LOD) was determined based on the standard deviation of the blank. The cut-off for LOD was three times the standard deviation of four blank runs.

## 3. Results

### 3.1. Theory of the Newton Photonics (NP) Waveguide-SPR Sensor

Traditional surface plasmon resonance is a technique for detecting changes in refractive index at the surface of a sensor. NP SPR measures the change of refractive index in the TM-polarized beam to a reference beam TE. The change in the refractive index is dependent on the mass of the analyte that is due to the binding or cleavage reaction on the chip surface. Since the mass accumulates at the sensor surface during a binding interaction, the refractive index increases and an increase in signal is observed. Change in the refractive index is measured by the interaction of the immobilized ligand and the flowed analyte on the sensor surface. The reference beam, TE, is generated by the same light source and propagated along the same path as the probe beam, but does not couple to the surface plasmon wave. As the surface-bound biomolecular mass changes, the SPR resonance curve starts to shift, causing a change in intensity of the TM beam while leaving the TE beam unaffected. The affinity of the analyte binding to the ligand at the sensor surface is directly proportional to the mass at the chip surface, leading to change in the refractive index at the TM beam when compared with the TE beam. This change in refractive index is measured in real time, and the result is plotted as refractive index units (RIU) versus time in a sensorgram. The RIU is an arbitrary unit. Therefore the polarity of a sensorgram from positive to negative can be changed easily. In this study, we have demonstrated the functioning of the SPR instrument and the detection of the BoNT/A light chain using SPR technology.

### 3.2. Performance of the NP Waveguide-SPR

**Figure 4:**
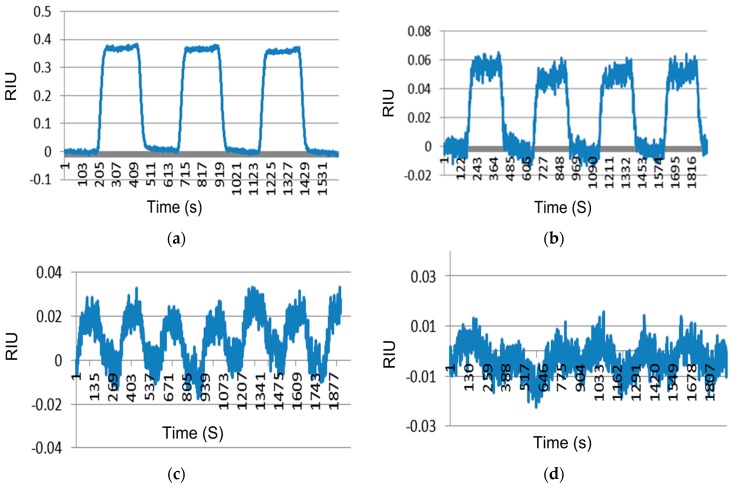
SPR sensorgrams of heavy water. SPR response to the concentration of D_2_O at (**a**) 2.2% of D_2_O as 100 refractive index units (RIU); (**b**) 0.22% of D_2_O as 10 RIU; (**c**) 0.022% of D_2_O as 1 RIU and (**d**) 0 of D_2_O (pure water) as a blank. X axis represents time in seconds and Y axis represents RIU signal in all the above sensorgrams.

SPR sensitivity is governed by the ability of the instrument to resolve change in the refractive index at the surface area. Various instruments measure the refractive index by an angular scan method and some by spectroscopic methods (measuring wavelengths) to determine the refractive index. The combination of the waveguide SPR approach with dual-polarization differential detection leads to a core sensitivity of 0.1 RIU (one RIU is a change of 10^−6^ in the refractive index) an improvement of 10-fold over the conventional angle-scan SPR approach (e.g., Biacore). The core sensitivity of our SPR sensor is demonstrated by detecting heavy water (D_2_O) mixed in regular water. While the refractive index of heavy water is 1.33 × 10^6^ RIU lower than the regular water, it is undifferentiated from regular water in all other chemical properties. Therefore, the SPR response to different concentrations of heavy water is a highly specific marker for the sensor’s core sensitivity. The test was performed by turning the heavy water sample flow through the chip’s flow cell on and off, and recording the resulting SPR signal. SPR response to the concentration of D_2_O at 2.2%, 0.22%, and 0.022%, corresponding to 100, 10 and 1 RIU, respectively, and compared to blank response (pure H_2_O), is indicated in the Figure 4.

### 3.3. BoNT/A LC Detection by NP Waveguide SPR System

**Figure 5:**
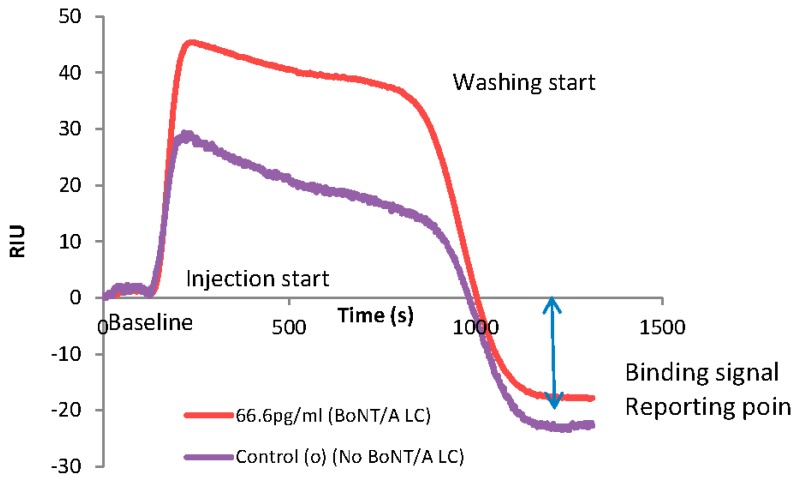
SPR sensorgram showed higher binding response units of uncleaved peptide as a control (purple) compared with a cleaved peptide (red) incubated with 666.6 pg/mL BoNT/A LC as a sample. The sensorgram demonstrated end of the sensorgram as a binding signal difference as a report point.

BoNT serotype A is the most toxic botulinum serotype with 50% of the lethal dose (LD_50_) being 0.1–1 ng/kg [28]. This study describes an attempt to develop an instrument and associated methods that can detect low amounts of BoNT rapidly. BoNT detection is accomplished by incubating the sample (spiked with BoNT/A LC) with the gold-labeled toxin-specific cleavage substrate and then injecting it into the SPR reader. The recording curve (sensorgram) for the detection of BoNT/A LC assay is shown in Figure 5. The mass difference of cleaved and uncleaved peptides generates different refractive indices compared with the reference-beam-generated SPR sensorgram. The SPR sensorgram from the NP-SPR exhibits baseline and analyte-to-ligand binding signal as a reporting point.

In the assay format for BoNT, the gold sensor chip was adsorbed with nutravidin, known to have higher affinity to biotin than the commonly used streptavidin. The analyte, made up of a BoNT endopeptidase peptide substrate, with biotin attached at the N-terminus and a 150 kDa Au nanoparticle attached at the C-terminus, was flowed over the Au chip surface coated with nutravidin (Figure 3). Upon incubation with different concentrations of BoNT/A LC, the serotype specific peptides were cleaved (Figure 3). The change in the mass of the cleaved and uncleaved peptides resulted in differential changes in the refractive index, thus leading to difference in the SPR signal.

Our goal was to develop the NP waveguide SPR system as a biosensor for rapid detection of biologically active BoNT. The BoNT activity was verified separately using a FRET (Forster resonance energy transfer) peptide substrate, as described previously [29]. In order to evaluate the sensitivity of the instrument, five different concentrations of BoNT/A LC were used in the range of 0 to 6.6 µg/mL, incubated with the gold-labeled toxin serotype specific cleavage substrate. The reaction mixture was then injected into the SPR reader. The higher concentrations of BoNT/A LC cleaved the peptide maximally, thus the signal was very low (less mass was captured by the sensor chip), compared to the control uncleaved substrate that resulted in maximum signal, as shown in a representative end of a sensorgram (Figure 5).

The Figure 5 is an example of a sensorgram recorded by the instrument as a plot of signal vs. time. The sensorgram illustrates a change in the slope at around 130 s as a start point of the injection that reflected a change of the mass transfer on the chip, leading to a change in the slope. Subsequently, at 730 s (which is 600 s after the start point; 400 µL sample at 40 µL/s loop) the washing buffer (PBS) reached the chip, resulting into a decrease in the SPR signal. The washing was complete within the next 480 s, reaching the reporting point of the bound peptide.

**Figure 6:**
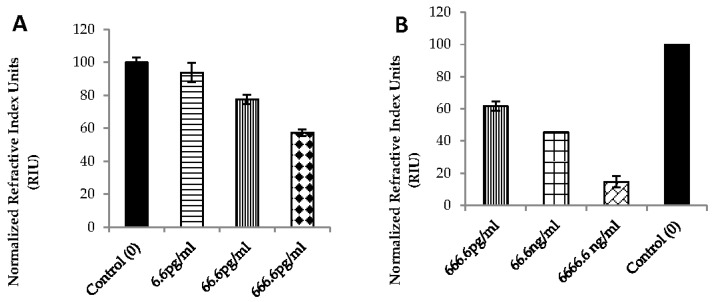
SPR sensorgrams of different BoNT/A LC concentrations incubated with peptide A in buffer, showing dose-dependent detection down to 6.6 pg/mL (**A**) and 666.6 pg/mL (**B**), recorded on two different chips.

The RIU is an arbitrary unit and the polarity of the sensorgram can easily be altered, so the negative sign in the RIU has been disregarded. The binding signal was measured using the steady state condition at the gold surface that is recorded as the reporting point in the sensorgram (Figure 5). Based on the maximum signal for the control uncleaved substrate, other cleaved signals were normalized to estimate relative cleavage. Variation in the signals at the reporting points of the sensorgrams indicated a dose dependent cleavage of the peptide (Figure 6). In order to prove the concept of using a waveguide SPR system as a biosensor for rapid detection of BoNT, we tested the biosensor over three log concentration ranges of BoNT/A LC. A direct binding assay by injecting sample through peptide A was rapidly detected in only 20 min with high sensitivity. The detection time including 30 min pre-incubation of BoNT/A LC with peptide will thus be less than 1 h. The sensor can detect up to 6.6 pg/mL BoNT/A LC (Figure 6). Different concentrations of the BoNT/A LC (from 6 pg/mL to over 6 µg/mL) were detected on a multichannel chip as a multiplex platform. The SPR signal was normalized using the negative control (no LC added) with uncleaved peptide (maximum mass) considered as 100% signal, shown as a bar graph in Figure 6A,B.

**Figure 7:**
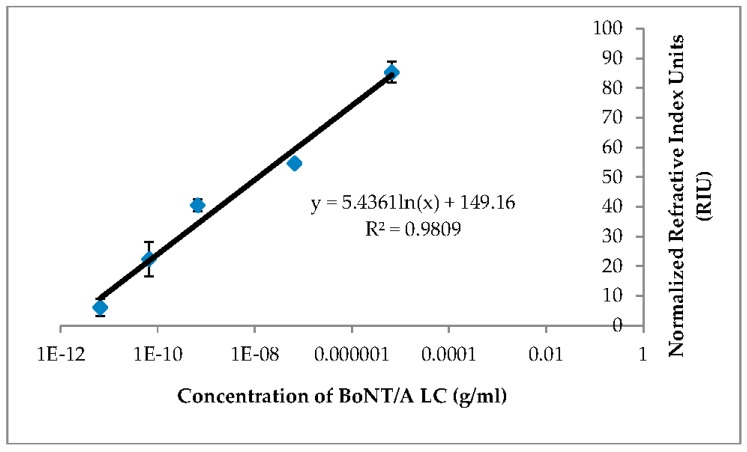
Detection of BoNT/A LC spiked in HEPES buffer. The data showed dose-dependent response of cleaved peptide. The result includes two independent runs.

The reproducibility of the signal was determined with 666.6 pg/mL concentration of BoNT/A LC sample. The logarithmic linearity of concentration to signal was demonstrated (R^2^ = 0.97, Figure 7) The LOD for BoNT/A LC is 6.76 ng/mL in buffer.

Similar data were produced while different concentrations of BoNT/B LC were incubated with substrate peptide B (Figure 3) derived from synaptobrevin, the natural substrate of BoNT/B (data not shown). The data indicated the reproducibility and reliability of NP SPR.

## 4. Discussion

We designed a new SPR biosensor using our proprietary waveguide technology, tested its utility for the rapid detection of enzymatically active botulinum neurotoxin, and demonstrated its feasibility as an assay for the rapid detection of botulinum neurotoxins. The detection limit was at least similar to, if not surpassing that of mouse bioassay (20–30 pg/mL for BoNT/A).

The serotype specific peptide with a higher specificity toward BoNT LCs makes a great tool for the rapid detection of enzymatically active toxin. While by itself LC is not toxic, it plays a critical role in the toxicity of BoNT. LC is the enzymatic domain that cleaves SNARE proteins, and makes the neurotoxin highly potent. This provides several advantages for using LC as a detection surrogate for BoNT toxicity, and for the development of rapid detection and diagnostic platforms for BoNT. In addition, due to the high selectivity of its targets, LC could also be used by bioterrorists using hybrid LC with other proteins as recognition domains, targeting other cells and tissues. Detection of LC would be the only approach for the surveillance of such new biothreats. We developed an NP sensor targeting BoNT LC, and demonstrated that it can rapidly detect BoNT/A LC with an equivalent sensitivity for BoNT that is comparable to mouse bioassay. Moreover, using LC to develop the rapid assay for BoNT does not require an expensive biosafety laboratory. Thus, we have developed a unique NP waveguide SPR, and have demonstrated the utility of the NP waveguide SPR as a biosensor for the rapid detection of active BoNT/A light chains. The limit of detection of the platform is 6.76 pg/mL, with a detection time of less than 20 min. While the analyte is the BoNT/A light chain, it is equivalent to 20 pg/mL of toxin (given that the molecular weight of LC is one third of toxin). This level of sensitivity is equivalent to approximately one mouse lethal dose (MLD) which is the LOD of the mouse bioassay. The run time includes an extensive washing step to identify the end reading point and excludes 30 min preincubation of BoNT/A LC with the peptide. Total detection time can be further reduced by optimizing the reaction condition, and by increasing the flow rate and terminating the session on the basis of a positive response after the sample flow time.

The NP SPR is capable of many different types of experiments, the technique demonstrated herein is applicable to multiple botulinum serotypes (other than A and B), and a wide array of other bioterror agents. The waveguide technology for NP SPR system has a different design from traditional SPR system (such as Biacore).

The NP SPR’s detection value was estimated by comparing the system response to regular pure water to that of varying concentrations of heavy water in regular water. Thus only the NP SPR system’s response to the refractive index was measured, without having to estimate the chemical efficiency of binding to the gold film, and thus the binding rate was calculated. Since the heavy water refractive index (1.3325) is known very precisely this approach is very clean and reliable. Even-though the same experiment was not performed in the Biacore system, its literature-based limit of quantification is 1 RU [30]. The observed 10-fold improved sensitivity is due to the following: (i) Each channel has its own reference signal and the ratio of the sample signal to the reference cancels all the common noise including the most important one, namely the coupling noise; (ii) The single mode operation is equivalent to use of the narrowest and most noiseless excitation angle in the Kretschmann configuration (Biacore machine).

Table 3 shows the main differences and similarities of the NP waveguide SPR and Biacore. NP SPR is better than the Biacore SPR in terms of sensitivity (Table 3). However, Biacore SPR has a much smaller sample loop than the NP SPR (60 nL vs. 1 µL). Using NP SPR, BoNT/A LC is detected up to 6.6 pg/mL. The LOD of BoNT/A LC is 6.76 ng/mL. The major advantage of the Biacore SPR is its software, because the instrument is already in the market and many scientists have worked with the software, making instrument data collection and analysis much easier, whereas the LAB view NP software is much more primitive at this stage. The NP SPR is only in a prototype form, requiring custom made chips, manual injection, and requires application-specific software development. The existing Biacore systems with similar features, like Biacore T100 or Biacore T200 systems, cost around $370K. In addition, Biacore dextrans chips cost $150 per chip. On the other hand, the cost of NP SPR even at the prototype level is less than 10% of the Biacore SPR cost and chips can be less than $2 with mass production, while the instrument provides similar sensitivity.

## 5. Conclusions

We developed a novel low-cost waveguide SPR sensor for rapid detection of botulinum neurotoxin. This unique biosensor system offers many advantages, including shorter detection time, label-free detection, small sample size, reusable sensor chips, the ability to handle complex samples, and real-time monitoring. We demonstrated the feasibility of an instrument and an assay for rapid detection of botulinum neurotoxins. Our waveguide NP SPR sensor showed the sensitivity comparable to the gold-standard mouse bioassay, with the limit of detection of 6.76 pg/mL for BoNT/A light chain. The NP SPR can be easily adapted into different types of experiments, and when combined with unique substrates of different serotypes of BoNT, it is capable for multiplex detection of other serotypes of BoNT, at a very affordable cost.

## Figures and Tables

**Table 1:** The NP SPR Instrument components and associated costs.

Instrument Parts	Estimated Cost
Laser and drive electronics	$800
Optical components (collimating lens, half waveplate, coupling prism, Wallaston prism)	$150
Fluidic cell	$20
Syringe pump and control system	$2000
Detection system (detector array, transimpedence amplifiers, data acquisition and processing)	$1000
Thermoelectric cooler	$120
Total cost	$4090

**Table 2:** SPR chip design parameters.

SPR Chip Design Parameters	
Substrate (lower cladding) index	1.4450
Guiding layer index	1.4559
Guiding layer thickness	2 µm
Chromium adhesion layer	2 nm
Gold refractive index	0.197 − 3.446i *
Gold pad thickness	35 nm

* i = √−1.

**Table 3:** Comparison of Biacore and NP SPR systems.

	Biacore SPR T100	NP Wave Guide SPR
Automated	Yes	Yes
Temperature Control (°C)	4–40	Room temperature
Flow Channels	4	4
Flow cell Volume	60 nL	1 µL
Refractive Index Range	1.33–1.36	Same
Detection principle	The molecular interaction (cleavage or binding) is detected by measuring the change in the resonance angle; SPR causes a reduction in the intensity of reflected light at a specific angle of reflection. This angle varies with the refractive index close to the surface on the side opposite from the reflected light (sample side).	The molecular interaction is detected by measuring the refractive index induced change in intensity of the TM-polarized component of a transmitted light beam. High sensitivity is achieved by using the index-insensitive TE-polarized component of the same light beam as a reference. Thus canceling out the instrument’s opto-mechanical noise
Software		LAB View ^TM^ (not material)
Baseline noise (RU)	1 resonance units (RU). (One RU = index change of 10^−6^	0.01 RU
Online subtraction of background response	Yes (Flow cells 2-1, 3-1,4-1)	Yes
Help to study	Affinity, Kinetics, Binding, Specificity, concentration	same
Instrumentation	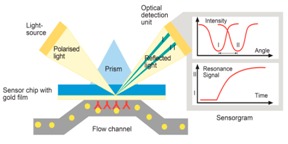	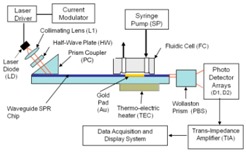
Sensor Chip Cost	$150 per chip for the dextran chips	<$2 (estimated)
